# Supplementary material for: Novel NMR Assignment Strategy Reveals Structural Heterogeneity in Solution of the nsP3 HVD Domain of Venezuelan Equine Encephalitis Virus
Source: Molecules. 2020 Dec 10;25(24):5824. doi: 10.3390/molecules25245824 (PMC7763327; doi:10.3390/molecules25245824)
Supplement: Supplementary file 1 [file molecules-25-05824-s001.pdf]

**(A)** aa-types: **S+1** / **S, S+1**

name of pulse sequences:
**music_ser_3d_sct2 / music_ser_3d_2_sct2** - semi-constant time in t2
**music_ser_3d_sct2si /music_ser_3d_2_sct2si** - semi-constant time in t2, sensitivity improvement
**music_ser_2d_trsctnd / music_ser_2d_2_trsctnd** - 2D semi-constant time TROSY

relevant acquisition parameters:
p24 = 1500us (for 600MHz and Q3.1000 pulse [sp9] )

cnst44 = 58.3 (adjusted to TMS scale)

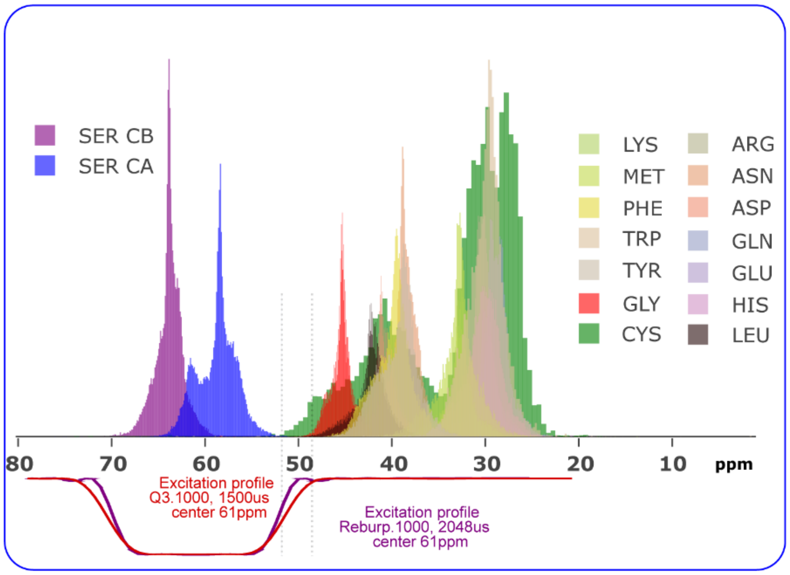

**(B)** pp-variants:
**music_lavia_3d_sct2 / music_lavia_3d_2_sct2** - semi-constant time in t2
**music_lavia_3d_sct2si /music_lavia_3d_2_sct2si** - semi-constant time in t2, sensitivity improvement
**music_lavia_2d_trsctnd / music_lavia_2d_2_trsctnd** - 2D semi-constant time TROSY

aa-types:
no option: **LA+1** / **LA, LA+1**
-DLABEL_VIA: **VIA+1** / **VIA, VIA+1**

relevant acquisition parameters:
p24 = 770us (for 600MHz and
Q3.1000 pulse [sp9,sp28] )
p33 = 570us (for 600MHz and
Q3.1000 pulse [sp23] )

cnst34 = 45.7 (adjusted to TMS scale)
cnst35 = 32.0 (adjusted to TMS scale)
cnst36 = 17.1 (adjusted to TMS scale)
cnst37 = 25.0 (adjusted to TMS scale)

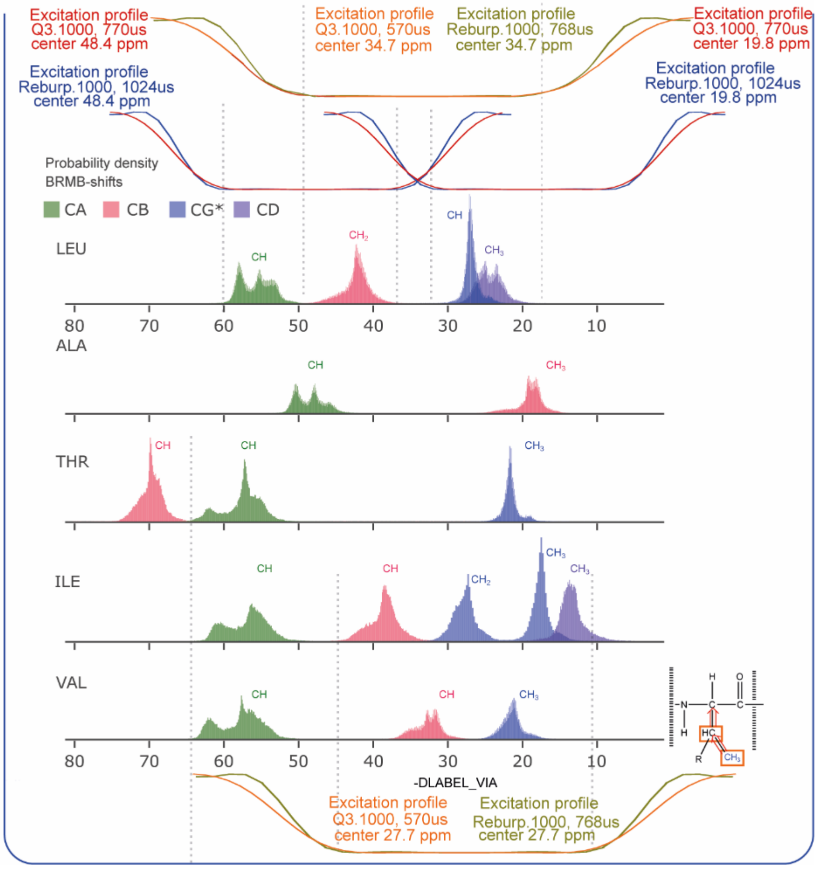

**(C)** pp-variants:

**music_pro_1_3d(.2)_sct2 / music_pro_2_3d(.2)_sct2** - semi-constant time in t2
**music_pro_1_3d(.2)_sct2si /music_pro_2_3d(.2)_sct2si** - semi-constant time in t2, sensitivity improvement
**music_pro_1_3d_trsctnd / music_pro_2_3d_trsctnd** - 3D semi-constant time TROSY
aa-types: **aa(Pro-1,P)** / **aa(Pro+1,P)**

relevant acquisition parameters:
**music_pro_2**: cnst22 = 61.0 (adjusted to TMS scale) and p24=770us for (600MHz) of Q3.1000 [sp9]
-------------------------
cnst26 = 101 ppm and p8 = 500us of Crp60,0.5,20.1 (default, N(Pro) decoupled from C,Cali)
or
cnst26 = 44 ppm (adjusted to TMS scale) and p8 = 256us of Q3.1000 (N(Pro) decoupled from Cali and $^{1}J(NC)$ appears)

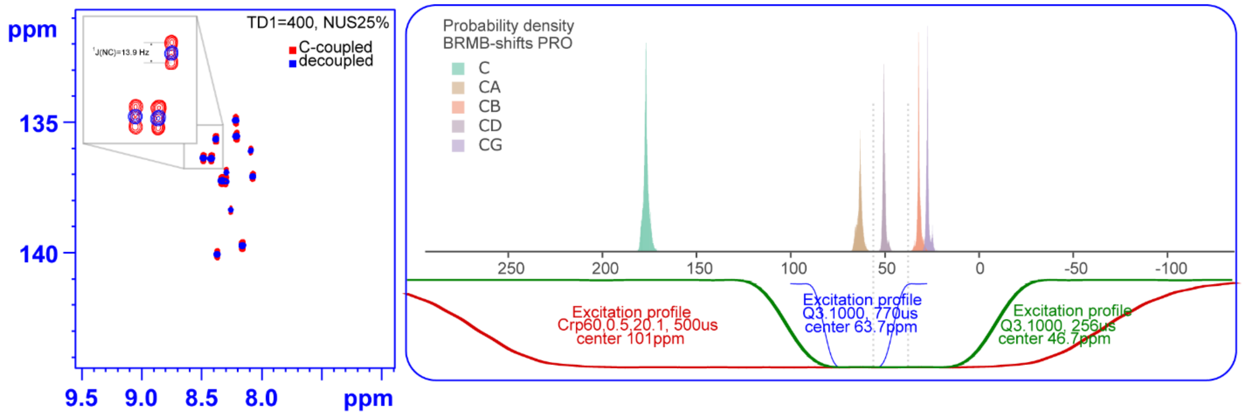


**(D)** pp-variants:

**music_kr_3d_sct2 / music_kr_3d_2_sct2** - semi-constant time in t2
**music_kr_3d_sct2si /music_kr_3d_2_sct2si** - semi-constant time in t2, sensitivity improvement
**music_kr_2d_trsctnd / music_kr_2d_2_trsctnd** - 2D semi-constant time TROSY

aa-types:
no option: **R+1** / **R, R+1**
-DLABEL_LYS: **KR+1** / **KR, KR+1**

relevant acquisition parameters:
p34 = 2000us (for 600MHz and Q3.1000 pulse [sp24,sp29] )

p35 = 3700us (for 600MHz and Q5.1000 pulse [sp25,sp26,sp27] )

cnst38 = 39.6 (adjusted to TMS scale)
cnst39 = 24.7 (adjusted to TMS scale)
cnst40 = 39.6 (adjusted to TMS scale)

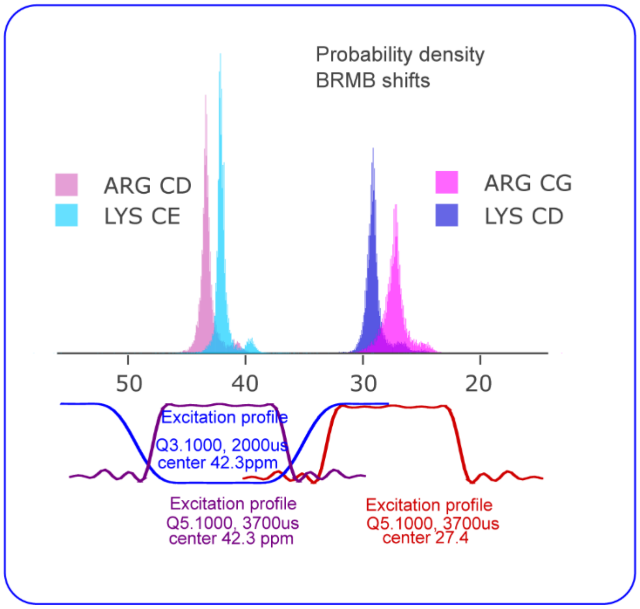

**(E)** pp-variants:

**music_ile_3d_sct2 / music_ile_3d_2_sct2** - semi-constant time in t2
**music_ile_3d_sct2si /music_ile_3d_2_sct2si** - semi-constant time in t2, sensitivity improvement
**music_ile_2d_trsctnd / music_ile_2d_2_trsctnd** - 2D semi-constant time TROSY

aa-types:
no option: **I+1** / **I, I+1**
possible breakthrough: **A**

relevant acquisition parameters:
p24 = 1300us (for 600MHz and
Reburp.1000 pulse [sp9] )
p34 = 2000us (for 600MHz and
Q3.1000 pulse [sp24,sp29] )

cnst37 = 24.9 (adjusted to TMS scale)
cnst45 = 9.4 (adjusted to TMS scale)
cnst46 = 31.3 (adjusted to TMS scale)

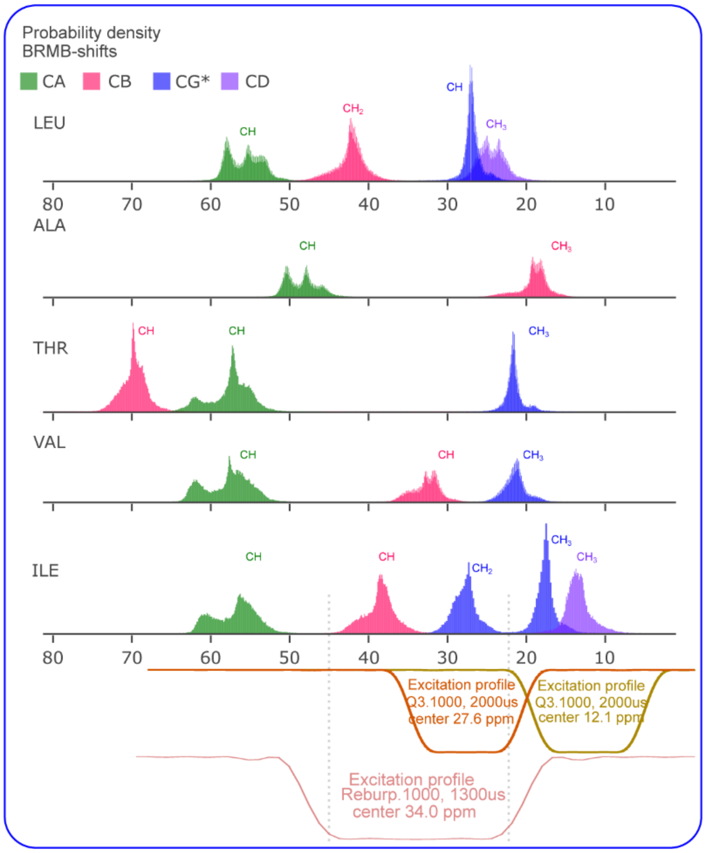

**(F)** pp-variants:
**music_fhyw_3d_sct2 / music_fhyw_3d_2_sct2** - semi-constant time in t2
**music_fhyw_3d_sct2si /music_fhyw_3d_2_sct2si** - semi-constant time in t2, sensitivity improvement
**music_fhyw_2d_trsctnd / music_fhyw_2d_2_trsctnd** - 2D semi-constant time TROSY

aa-types:
no option: **FHY+1** / **FHY, FHY+1**
(or **FHYW+1** / **FHYW, FHYW+1**
see below)
-DLABEL_TRP: **W+1** / **W, W+1**

relevant acquisition parameters:
p23 = 1400us (for 600MHz and
Q5.1000 pulse [sp10,sp12] )

cnst41 = 129.5 (adjusted to TMS scale)
cnst42 = 101.4 (adjusted to TMS scale)

for **FHYW+1** / **FHYW, FHYW+1**
p23 = 770us (for 600MHz and
Q5.1000 pulse [sp10,sp12] )

cnst41 = 120.4 (adjusted to TMS scale)

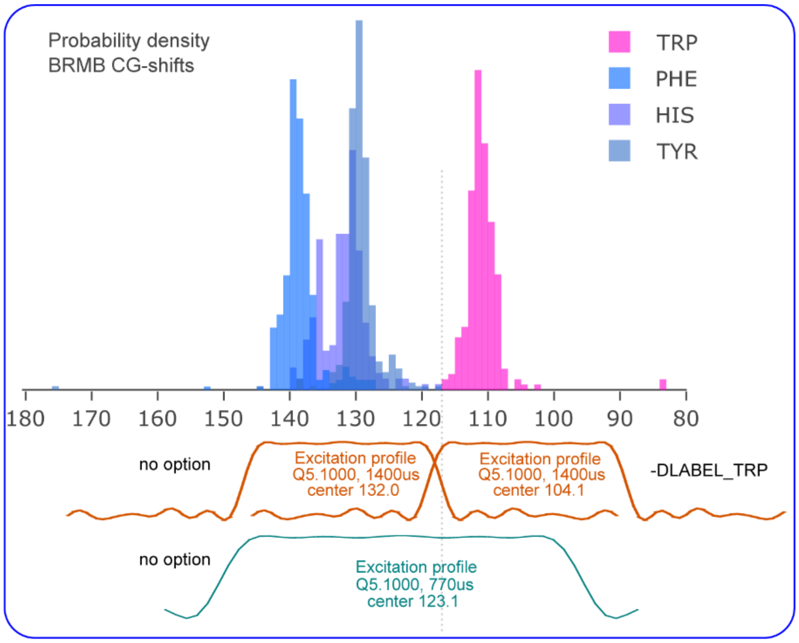


**(G)** pp-variants:
**music_de_3d_sct2 / music_de_3d_2_sct2** - semi-constant time in t2
**music_de_3d_sct2si /music_de_3d_2_sct2si** - semi-constant time in t2, sensitivity improvement
**music_de_2d_trsctnd / music_de_2d_2_trsctnd** - 2D semi-constant time TROSY

aa-types:
no option: **D+1** / **D, D+1**
-DLABEL_GLU: **E+1** / **E, E+1**
possible breakthrough: **G**, **D** (in **E**)

relevant acquisition parameters:
p24 = 1500us (for 600MHz and
Q3.1000 pulse [sp9] )

cnst38 = 37.0 (adjusted to TMS scale)

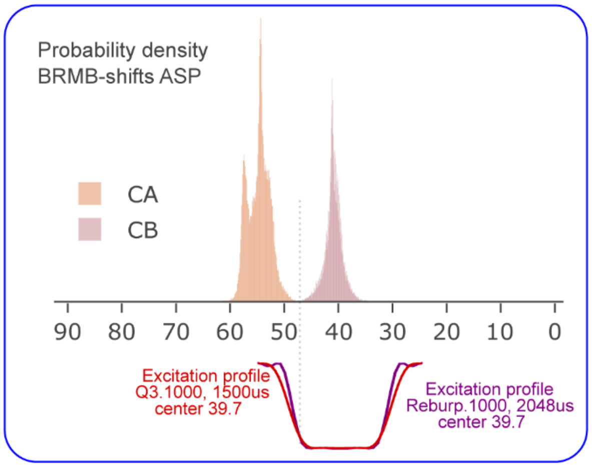

**(I)**

pp-variants:
**music_tavi_3d_sct2 / music_tavi_3d_2_sct2** - semi-constant time in t2
**music_tavi_3d_sct2si /music_tavi_3d_2_sct2si** - semi-constant time in t2, sensitivity improvement
**music_tavi_2d_trsctnd / music_tavi_2d_2_trsctnd** - 2D semi-constant time TROSY

aa-types:
no option: **TAVI+1** / **TAVI, TAVI+1**
-DLABEL_ALA: **A** / **A, A+1**
-DLABEL_THR: **TA+1** / **TA, TA+1**

relevant acquisition parameters:
for -DLABEL_THR
p24 = 1024us (for 600MHz and
Reburp.1000 pulse [sp9] )

cnst43 = 62.0 (adjusted to TMS scale)

In fact, spectrum of
music_tavi (with no option) >=
music_tavi (-DLABEL_THR) +
music_lavia (-DLABEL_VIA)
(they are its sub-spectra)

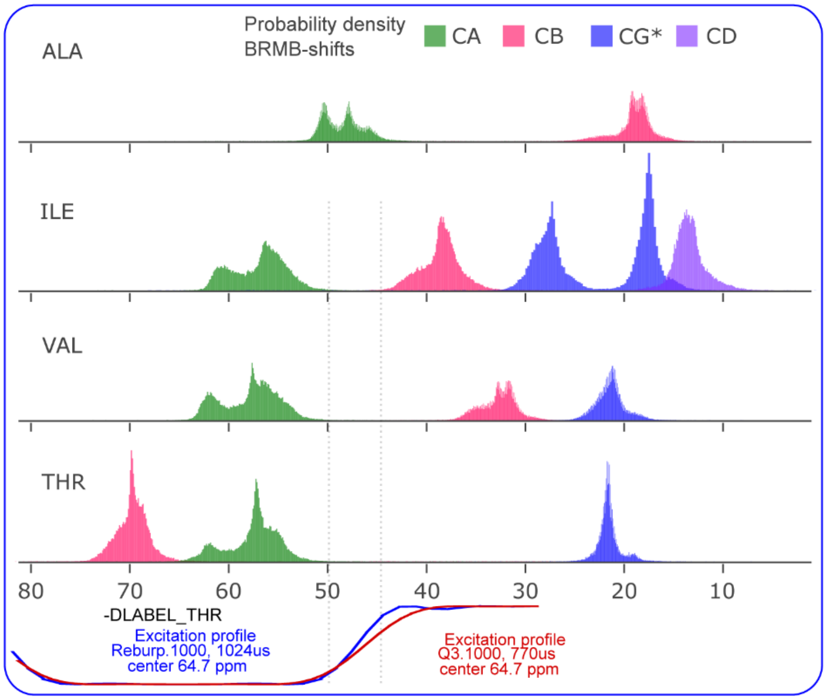


Fig. S1 Pulse programs information include aa-type of MUSIC experiments with parameters adjusted for 600MHz spectrometer: (A) S+1/S, S+1; (B) LA+1/LA, LA+1 and VIA+1/VIA,VIA+1; (C) PRO; (D) R+1/R, R+1 and KR+1/ KR, KR+1; (E) I+1/I, I+1; (F) FHY+1/FHY, FHY+1 and W+1/W,W+1; (G) D+1/D, D+1 and E+1/E, E+1; (I) TAVI+1/TAVI, TAVI+1 or A+1/A, A+1 and TA+1/TA, TA+1 where a capital symbols are the short abbreviation of aa type. All Panels contain the figures ( in square boxes) showing the probability density of carbons nuclei belonging to the different type of aa based on Biological Magnetic Resonance Data Bank (BMRB) data set. It is also presented the regions of excitation in $^{13}$C spectra performed by $^{13}$C band-selective Q3 and Q5 (or time reversed Q5)(Emsley & Bodenhausen, 1992) or Reburp(Geen & Freeman, 1991) shapes pulses including its durations and the centre of excited region. The name of pulse sequences and values of constants used in each experiments (adjusted to TMS scale) are listed in every Panels.

## Literature

Emsley, L. & Bodenhausen, G. (1992). *J Magn Reson* **97**, 135-148.
Geen, H. & Freeman, R. (1991). *J Magn Reson* **93**, 93-141.
